# Poly-L-lysine Prevents Senescence and Augments Growth in Culturing Mesenchymal Stem Cells* Ex Vivo*

**DOI:** 10.1155/2016/8196078

**Published:** 2016-06-15

**Authors:** June Seok Heo, Hyun Ok Kim, Seung Yong Song, Dae Hyun Lew, Youjeong Choi, Sinyoung Kim

**Affiliations:** ^1^Cell Therapy Centre, Severance Hospital, Seoul 03722, Republic of Korea; ^2^Department of Laboratory Medicine, Yonsei University College of Medicine, Seoul 03722, Republic of Korea; ^3^Department of Plastic and Reconstructive Surgery, Yonsei University College of Medicine, Seoul 03722, Republic of Korea

## Abstract

Mesenchymal stem cells (MSCs) possess great therapeutic potential. Efficient* in vitro* expansion of MSCs is however necessary for their clinical application. The extracellular matrix (ECM) provides structural and biochemical support to the surrounding cells, and it has been used as a coating substrate for cell culture. In this study, we have aimed to improve the functionality and stemness of MSCs during culture using poly-L-lysine (PLL). Functionality of MSCs was analysed by cell cycle analysis, differentiation assay, β-galactosidase staining, and RT-PCR. Furthermore, we assessed the global gene expression profile of MSCs on uncoated and PLL-coated plates. MSCs on PLL-coated plates exhibited a faster growth rate with increased S-phase and upregulated expression of the stemness markers. In addition, their osteogenic differentiation potential was increased, and genes involved in cell adhesion, FGF-2 signalling, cell cycle, stemness, cell differentiation, and cell proliferation were upregulated, compared to that of the MSCs cultured on uncoated plates. We also confirmed that MSCs on uncoated plates expressed higher β-galactosidase than the MSCs on PLL-coated plates. We demonstrate that PLL provides favourable microenvironment for MSC culture by reversing the replicative senescence. This method will significantly contribute to effective preparation of MSCs for cellular therapy.

## 1. Introduction

The differentiation of mesenchymal stem cells (MSCs) into multiple cell lineages can be exploited as an attractive strategy for cell-based therapy and regenerative medicine [1]. MSCs can easily be obtained from various human tissue sources such as the bone marrow, cord blood, placenta, and adipose [2–5]. The clinical application of MSCs to tissue engineering has been introduced due to their numerous advantages including high expansion potential and extensive differentiation potential [6, 7]. However, MSCs need to be expanded* in vitro* in order to obtain sufficient cells for clinical trials since they are extremely rare in various tissues. Unlike embryonic stem cells, adult stem cells (MSCs) have a limited lifespan and stop proliferating during* in vitro* culture due to replicative senescence [8].

Cellular senescence, which is morphologically characterized by an enlarged and flattened cell shape, was first described by Hayflick [9]. Cellular senescence refers to active cells that eventually enter a state of irreversible growth arrest. Moreover, replicative senescence of MSCs exhibits reduced functionality, and cellular senescence might impair the regenerative potential of MSCs [10]. Studies investigating MSC senescence are therefore crucial for successful therapeutic application of MSCs. The mechanisms underlying the cellular senescence of MSCs are still poorly understood. Studies show that replicative senescence or cellular senescence is induced by intrinsic or extrinsic environmental factors [11]. The shortening of telomeres constitutes an intrinsic factor, whereas DNA damage is considered an extrinsic factor. Specifically, oxidative stress by reactive oxygen species (ROS) is the main extrinsic factor that induces senescence [12]. Cellular senescence is a complex process, and its molecular mechanisms are unknown. A number of studies demonstrated that hypoxia is beneficial to the senescence of MSC; however the precise understanding mechanism is not clear [13–15]. It was also shown that inhibition of the p16 tumour suppressor gene delays growth arrest and therefore senescence of MSC [16]. Additionally, Abedin and King showed that FGF-2 suppresses the cellular senescence of human MSCs [17]. It is hard to preserve the important characteristics such as proliferation capacity and stemness of MSCs the inadequate cultivating microenvironment* in vitro*. Therefore, establishing an optimized culture condition that delays the senescence of MSCs is imperative.

MSCs naturally reside in a specialized niche* in vivo*, which mainly consists of the extracellular matrix (ECM). The ECM provides structural and biochemical support to the cells and has various other functions including cell adhesion, cell to cell communication, and differentiation [17, 18]. Poly-L-lysine (PLL) of extracellular matrix proteins is a small natural homopolymer of the essential amino acid L-lysine that is used to coat culture substrates. PLL functions as an attachment factor that enhances cell adherence due to its strong affinity for proteins and electrostatic interactions between the positive charges on the PLL molecule and the negative charges on the cell membrane [19, 20]. Park et al. showed that PLL increases the* ex vivo* expansion and erythroid differentiation of human hematopoietic stem cells [21]. It was also reported that PLL promoted neural progenitor cell function, and it is commonly used for MSC differentiation into neural lineages [22]. Recent studies suggest that neuroectodermal cells can generate MSCs, and they may arise in the neural crest, which is derived from embryonic neuroectoderm [23, 24]. These studies emphasized the interesting possibility that PLL could provide a favourable environment for MSC culture* in vitro*. We therefore hypothesized that PLL would be beneficial for MSC culture expansion and would preserve MSC properties* in vitro*. In this study, PLL-coated plates were used for MSC culture expansion. To the best of our knowledge, this study is the first to compare genome-wide expression profiles of MSCs cultured on PLL-coated plates with MSCs cultured on uncoated plates. In addition, we compared and analysed properties of MSCs cultured on PLL-coated plates with uncoated plates. The PLL-coated surface provided an excellent environment that improved the stemness of MSCs and delayed their senescence through upregulation of genes involved in cell adhesion, FGF-2 signaling, cell cycle, stemness, cell differentiation, and proliferation. This method could be useful for* in vitro* expansion of highly functional MSCs for cell-based therapeutic applications.

## 2. Materials and Methods

### 2.1. Reagents

Dulbecco's Modified Eagle Medium (DMEM), *α*MEM, foetal bovine serum (FBS), penicillin/streptomycin (P/S), 0.4% trypan blue stain, and TRIzol were obtained from Gibco (Invitrogen, Carlsbad, CA, USA). Mesenchymal stem cell growth medium (MSCGM), osteogenic differentiation medium, adipogenic differentiation medium, and chondrocyte differentiation medium were obtained from Cambrex (Lonza, Allendale, NJ, USA). Poly-L-lysine (PLL) and propidium iodide (PI) were purchased from Sigma-Aldrich (St. Louis, MO, USA). The senescence detection kit was obtained from BioVision Inc. Oligonucleotides for polymerase chain reaction (PCR), reverse transcription, and cDNA were synthesized by Bioneer (Bioneer Corporation, Daejeon, Korea). Silver nitrate, oil red O, and safranin O for differentiation staining were purchased from Sigma-Aldrich.

### 2.2. Cell Culture

MSCs were isolated from human bone marrow as previously described [25]. Cells after informed consent were collected from healthy three donors with approval from the Research Ethics Committee of Severance Hospital (Approval number 4-2014-0650). Primary cells of passage 0 were cultured and maintained in low glucose DMEM (DMEM-LG) supplemented with 10% FBS and 1% P/S at 37°C in 5% CO_2_. Cells were harvested using 0.05% trypsin/EDTA (Invitrogen) when they reached 80–90% confluence for further experiment. Harvested 2 × 10^4^ cells/well in 12-well plates were replated in 0.01% PLL-coated or uncoated plates for all experiments.

### 2.3. Flow Cytometry Analysis

For immunophenotyping, MSCs were stained with fluorescein isothiocyanate- (FITC-) or phycoerythrin- (PE-) conjugated monoclonal antibodies: CD14-FITC, CD29-FITC, CD31-PE, CD34-FITC, CD44-PE, CD45-PE, CD73-PE, CD90-FITC, CD105-PE, and CD106-FITC (all from BD Pharmingen, San Diego, CA, USA). Additionally, FITC- and PE-conjugated isotype controls were used as negative controls. Briefly, cultured MSCs were harvested and stained with the antibodies for 20 min at 4°C. Subsequently, the stained cells were washed with phosphate buffered saline (PBS) and fixed with 1% paraformaldehyde (Biosesang, Seongnam, Korea). The cells were analysed using a flow cytometer (Cytomics Flow Cytometer; Beckman Coulter, Fullerton, CA, USA).

### 2.4. Growth Characteristics

For analysis of cell proliferation, MSCs were plated at a density of 2 × 10^4^ per well in uncoated or PLL-coated 12-well plates (Corning Inc., Corning 07-200-81, NY, USA) in MSC culture medium. Cultures were maintained for 5 days and then harvested for cell counting on days 3, 4, and 5. The proliferation rate of cells was determined using the trypan blue exclusion method. The population doubling time was calculated as the cumulative number of serial cells passaging until the cells reached senescence [26]. At passage 7, cells were photographed.

### 2.5. Cell Cycle Analysis

The cultured MSCs at passage 7 were removed from uncoated and PLL-coated plates. Harvested cells washed with PBS and fixed with cold 70% ethanol while minimizing clumping. After 30 min at 4°C, the cells were washed with PBS and stained with propidium iodide. Propidium iodide fluorescence was then examined using the Cytomics Flow Cytometer (Beckman Coulter).

### 2.6. *β*-Galactosidase Staining

*β*-galactosidase staining was performed using the senescence associated *β*-galactosidase staining kit (BioVision Inc.), according to the manufacturer's instructions. Briefly, passage 7 MSCs cultured on uncoated and PLL-coated plates were washed with PBS and fixed with 4% paraformaldehyde at room temperature. After washing with PBS, cells were incubated with senescence-associated *β*-galactosidase (SA-*β*-gal) staining solution for 24 h at 37°C. The number of *β*-galactosidase positive cells (blue colour) was evaluated under a light microscope (Olympus-IX71), as an indicator of the number of senescent cells.

### 2.7. Reverse Transcription PCR (RT-PCR)

Total RNA was prepared using TRIzol reagent, and cDNA was synthesized using transcriptase II (Invitrogen). RT-PCR was performed with PCR primers under the conditions listed in Table 1 (Bioneer). Glyceraldehyde 3-phosphate dehydrogenase (GAPDH) was used as an internal standard. The signal intensity of the product was normalized to its respective GAPDH signal intensity.

### 2.8. Differentiation Assay

To assess the differentiation potential of MSCs, cells were seeded at 7 × 10^4^/well in 12-well plates for the induction of osteogenesis and chondrogenesis and 1.5 × 10^5^/well in 12-well plates for inducing adipogenesis. For differentiation, primary and passage 7 MSCs were maintained for 14 days in osteogenic, adipogenic, or chondrogenic differentiation medium (Lonza). For chondrogenesis, cells were treated with 10 ng/mL TGF- (transforming growth factor-) *β*3 (Lonza). After induction, Von Kossa staining was applied to analyse osteogenic differentiation, and calcium content was evaluated using the calcium (CPC) liquicolor kit (Stanbio Laboratory, Boerne, USA), based on a previously reported method [27]. Briefly, the cells were washed with PBS and treated 0.5 N HCl. After shaking for 3 h with an orbital shaker, the supernatant was transferred to a new tube for analysis. Ortho-cresolphthalein complexone (OCPC) was added to the sample, and absorbance was detected at 550 nm. After adipogenic differentiation, lipid droplets were detected by oil red O staining, and absorbance was measured at 500 nm after destaining with isopropanol for 30 min for quantitative analysis according to the previously reported method [27]. To evaluate chondrogenesis, cells were stained with safranin O solution, and the absorbance of sulphated glycosaminoglycan was detected at 656 nm using the Blyscan assay kit (Biocolor Ltd.) for quantitative analysis. Briefly, the supernatant of each sample was mixed with DMMB dye and reagents according to the manufacturer's protocols and reference [28]. Experiments were performed in triplicate.

### 2.9. Human Genome Microarray

The synthesis of target cRNA probes and hybridization were performed using Agilent's Low RNA Input Linear Amplification kit (Agilent Technology, USA), according to the manufacturer's instructions. Briefly, 1 *μ*g of total RNA and T7 promoter primers were mixed and incubated at 65°C for 10 min. The cDNA master mix (5x first strand buffer, 0.1 M DTT, 10 mM dNTP mix, RNase-Out, and MMLV-RT) was prepared and added to the reaction mix. The samples were incubated at 40°C for 2 h and were then incubated at 65°C for 15 min to terminate RT and dsDNA synthesis. The transcription master mix was prepared according to the manufacturer's protocol (4x transcription buffer, 0.1 M DTT, NTP mix, 50% PEG, RNase-Out, inorganic pyrophosphatase, T7-RNA polymerase, and cyanine 3/5-CTP). Transcription of dsDNA was performed by adding the transcription master mix to dsDNA reaction samples and incubating at 40°C for 2 h. Amplified and labelled cRNA was purified using the cRNA Cleanup Module (Agilent Technology), according to the manufacturer's protocol. The labelled cRNA target was quantified using a ND-1000 spectrophotometer (NanoDrop Technologies, Inc., Wilmington, DE). After checking the labelling efficiency, fragmentation of cRNA was performed by adding 10x blocking agent and 25x fragmentation buffer and incubating at 60°C for 30 min. The fragmented cRNA was resuspended with 2x hybridization buffer and directly pipetted onto assembled Agilent's Human Oligo Microarray (44 K). The arrays hybridized at 65°C for 17 h using an Agilent hybridization oven (Agilent Technology, USA). The hybridized microarrays were washed, according to the manufacturer's washing protocol (Agilent Technology, USA).

### 2.10. Data Acquisition and Analysis

All data normalization and selection of differentially expressed genes were performed using GeneSpringGX 7.3 (Agilent Technology, USA). The averages of normalized ratios were calculated by dividing the average normalized signal channel intensity by the average normalized control channel intensity. Functional annotation of genes was performed according to the Gene Ontology*™* Consortium (http://www.geneontology.org/index.shtml) by GeneSpringGX 7.3. Gene classification was based on searches of the BioCarta (http://www.biocarta.com/), GenMAPP (http://www.genmapp.org/), DAVID (http://david.abcc.ncifcrf.gov/), and Medline databases (http://www.ncbi.nlm.nih.gov/).

### 2.11. Statistical Analysis

Statistical analysis was performed using Student's* t*-test. Quantitative data are expressed as means ± SD. Differences are considered statistically significant at *p* < 0.05.

## 3. Results

### 3.1. Characterization of Cultured MSCs

**Figure 1:**
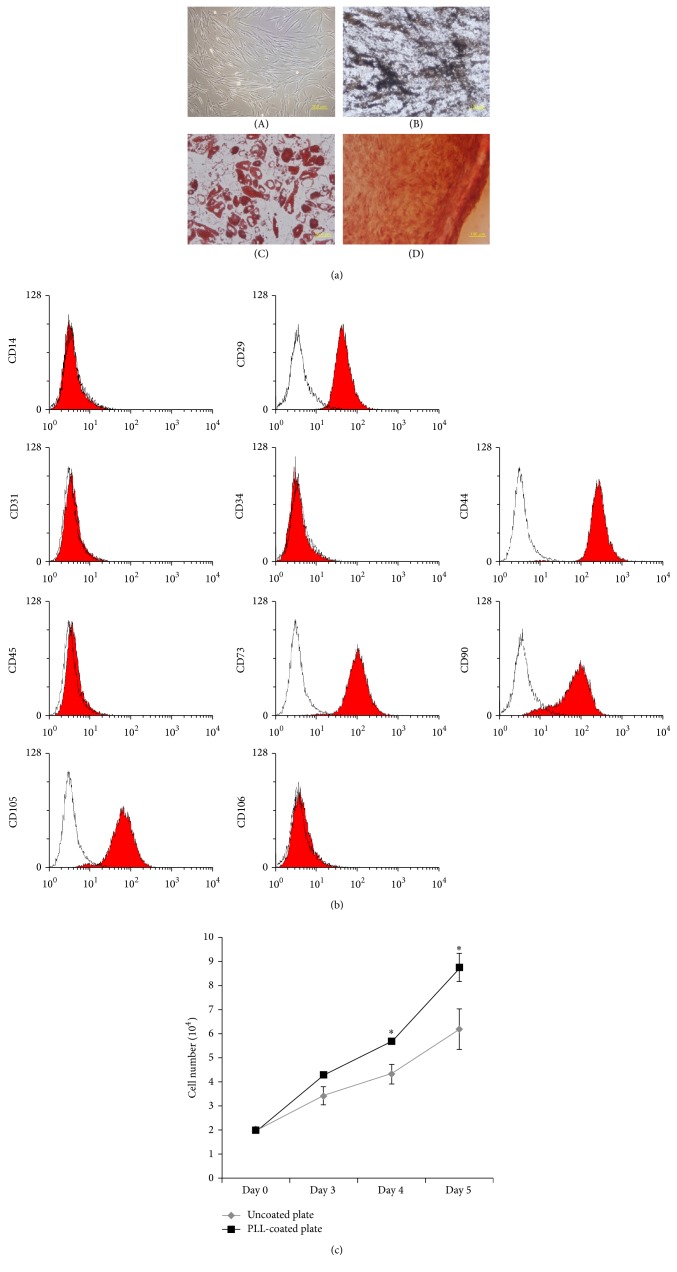
Characteristics and short-term culture of MSCs. (a) Cell morphology was observed under phase-contrast microscopy ((A) magnification: 100x) and differentiation potential was evaluated by Von Kossa, oil red O, and safranin O staining ((B) osteogenesis-magnification: 200x, (C) adipogenesis-magnification: 400x; (D) chondrogenesis-magnification: 200x). (b) The immunophenotype of bone marrow-derived MSCs. Flow cytometry histograms show that cultured MSCs were positive CD29, CD44, CD73, CD90, and CD105. These results show representative histograms of cultured MSCs. (c) Proliferative activity of cultured MSCs. MSCs were cultured on uncoated or poly-L-lysine- (PLL-) coated plates for 5 days. The number of harvested cells was measured by trypan blue staining. The data represent the mean ± standard deviation of three independent experiments (*n* = 3). ^*∗*^ *p* < 0.05.

MSCs were isolated and cultured from human bone marrow of three different donors. Cultured MSCs displayed a fibroblast-like morphology, and they were differentiated into osteocyte, chondrocyte, and adipocyte under proper conditions (Figure 1(a)). For immunophenotyping of cultured MSCs, MSCs derived from different donors were analysed by flow cytometry.  Figure 1(b) shows that MSCs were positive for MSC markers, including CD29, CD44, CD73, CD90, and CD105, whereas MSCs were negative for CD14, CD31, CD34, CD45, and CD106 known as hematopoietic and endothelial markers. The results of flow cytometry demonstrate that the cultured cells were typical MSCs.

### 3.2. Growth Kinetics of Cultured MSCs on Uncoated and PLL-Coated Plates

To determine the effect of PLL on* in vitro* culture of MSCs, we compared the proliferation activity of MSCs cultured on uncoated and PLL-coated plates. The optimal concentration of 0.01% PLL used for this study was determined empirically, since we observed that high concentrations greater than 0.01% hindered MSC adhesion and spread (data not shown). Bone marrow-derived MSCs at passage 3 were cultured for 5 days to determine whether PLL stimulates MSC proliferation in the short-term culture. The numbers of harvested cells at days 3, 4, and 5 were measured using the trypan blue exclusion method. An increase in cell numbers was detected in MSCs cultured on PLL-coated plates compared to cells grown in the absence of PLL-coating (Figure 1(c)).

### 3.3. PLL Suppressed MSC Replicative Senescence

**Figure 2:**
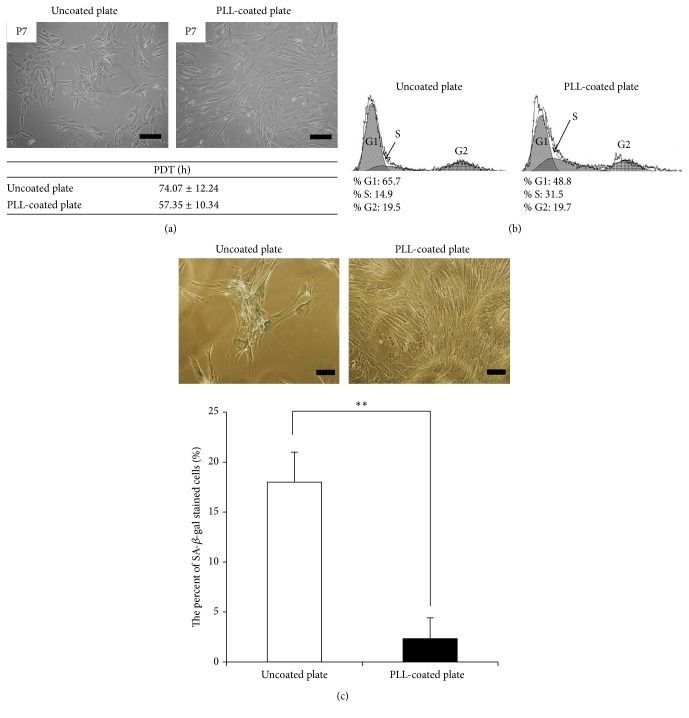
Changes in senescent cells induced by extracellular matrix (ECM) coating. (a) Morphological changes and population doubling time in senescent cells cultivated on a poly-L-lysine- (PLL-) coated plate were observed compared to cells cultured on an uncoated plate (magnification: 100x, scale bar = 200 *μ*m). (b) Cell cycle analysis. Cells were removed from the culture well, stained for DNA with propidium iodide (PI), and analysed by flow cytometry. (c) Senescence associated *β*-gal assay of MSCs cultured on uncoated and PLL-coated plates. One representative of three independent experiments is shown. The number of *β*-gal positive cells was enumerated. The data represent the mean ± standard deviation of three experiments (magnification: 200x, scale bar = 100 *μ*m). ^*∗∗*^ *p* < 0.01.

Passage 7 MSCs cultured in DMEM on uncoated plates displayed the typical phenomenon of replicative senescence with morphological abnormalities (Figure 2(a)). MSCs are typically cultivated in DMEM-LG containing 10% FBS and 1% P/S. Additionally, *α*MEM and mesenchymal stem cell growth medium (MSCGM) are also used for MSC culture. MSCGM is a known specific medium for mesenchymal stem cell growth. To investigate morphological changes of replicative senescence of MSCs, cells were replated in *α*MEM and MSCGM with DMEM. MSCs grown in *α*MEM and MSCGM exhibited similar morphological patterns including cell aggregation as MSCs grown in DMEM (data not shown). Senescent cells were subsequently seeded on uncoated and PLL-coated plates to determine whether PLL could induce any changes during culture. Surprisingly, MSCs seeded on uncoated plates maintained an aggregated phenotype with growth arrest, whereas cells on the PLL-coated plates demonstrated an increased growth rate without aggregation (Figure 2(a)). The population doubling time of MSCs cultured on PLL-coated plates was decreased compared with that of MSCs on uncoated plates in culture (Figure 2(a)). Additionally, MSCs cultured on uncoated plates did not demonstrate further growth; however MSCs cultured on PLL-coated plates reached 100% confluence within several days. Furthermore, to examine whether PLL increased the S-phase, cell cycle analysis was carried out using flow cytometry. PLL increased the S-phase of MSCs compared to MSCs cultured in the absence of PLL (Figure 2(b)). We next investigated whether PLL suppressed MSC senescence. MSCs cultured on uncoated and PLL-coated plates were stained with *β*-galactosidase after the typical phenomenon of senescence was observed. MSCs cultured on uncoated plates showed a significant increase in the percentage of *β*-galactosidase positive cells compared to cells cultured on PLL-coated plates (Figure 2(c)).

### 3.4. PLL Induced Stemness Markers and Inhibited Senescence Related Genes

**Figure 3:**
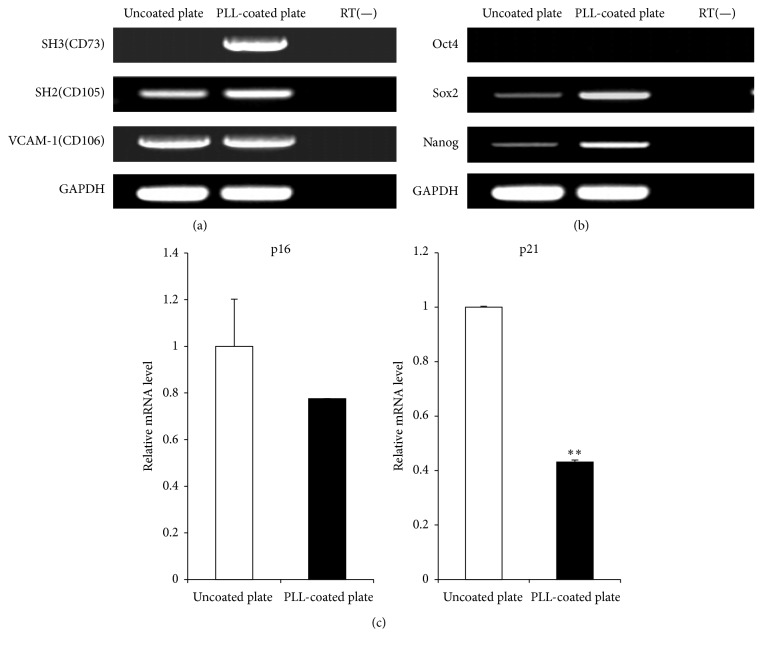
Gene expression of cells cultured on uncoated and poly-L-lysine- (PLL-) coated plates. Gene expression was analysed by RT-PCR for (a) MSC and (b) stemness markers. (c) p16^INK4a^ and p21^Cip1^ mRNA expression levels were evaluated using RT-PCR. Expression level relative to that of housekeeping gene GAPDH is shown. ^*∗∗*^ *p* < 0.01.

The gene expressions of known MSC markers* CD73*,* CD105*, and* CD106* in MSCs at passage 7 showing a senescent phenotype cultured on uncoated and PLL-coated plates were analysed using RT-PCR analysis. All MSCs, regardless of cell culture conditions, were positive for* CD105* and* CD106* (Figure 3(a)). Importantly, very low levels of* CD73* (ecto-5′-nucleotidase), a MSC specific marker, were detected in senescent MSCs cultured on uncoated plates, whereas it was distinctly expressed in MSCs cultured on PLL-coated plates (Figure 3(a)).* Oct4*,* Nanog,* and* Sox2* that regulate the maintenance of the pluripotency have been purported to play a similar role also in mesenchymal stem cells [29, 30]. To determine whether stemness was affected by PLL, we investigated the gene expression levels of stemness markers such as* Oct4*,* Sox2*, and* Nanog*.* Oct4* was undetectable in all MSCs regardless of culture conditions. However* Sox2* and* Nanog* were upregulated in MSCs cultured on PLL-coated plates compared to senescent MSCs cultured on uncoated plates (Figure 3(b)). These results indicate that MSC and stemness markers were affected by PLL. In addition, to confirm whether PLL affected cell proliferation, p16^INK4a^ and p21^Cip1^ genes associated senescence were analysed by RT-PCR. As shown in Figure 3(c), the mRNA expression levels of p16^INK4a^ and p21^Cip1^ were decreased in cells cultured on PLL-coated plates compared to the cells on uncoated plates. In particular, the decreasing of p21 is statistically significant in passage 7 of PLL-coated plate (Figure 3(c)). These results demonstrate that senescent MSCs can be cultured normally on PLL-coated plates and that PLL can inhibit MSC-replicative senescence.

### 3.5. Differentiation Potential of MSCs Cultured on PLL-Coated Plates

**Figure 4:**
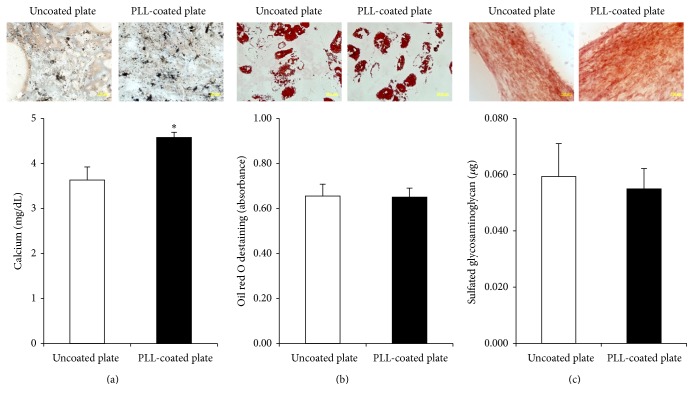
Differentiation potential of cells cultured on uncoated and poly-L-lysine- (PLL-) coated plates. (a) Osteogenesis was determined by Von Kossa staining and calcium quantification. (b) Adipogenesis was examined by oil red O staining. For quantitative analysis, absorbance was detected after destaining. (c) Chondrogenesis was analysed by safranin O staining and glycosaminoglycan quantification. The data represent the mean ± standard deviation of three independent experiments. ^*∗*^ *p* < 0.05.

To assess the differentiation potential of MSCs, cells at passage 7 showing a senescent phenotype were cultured and induced to differentiate to osteocytes, adipocytes, and chondrocytes in specific differentiation media. Von Kossa staining was used to detect calcium-containing mineralized nodules indicating osteogenic induction. The results demonstrated that MSCs cultured on PLL-coated plates showed higher amounts of Von Kossa staining compared to cells on uncoated plates, and MSCs cultured on PLL-coated plates had higher calcium accumulation compared to cells on uncoated plates in the calcium content assay (Figure 4(a)). Adipogenic differentiation was analysed by oil red O staining. Both MSCs cultured on uncoated and PLL-coated plates showed lipid droplet formation, and the absorbance value for oil red O staining was similar between the two culture conditions (Figure 4(b)). The chondrogenic differentiation potential of MSCs was assessed using safranin O staining. Similar to the results of adipogenic differentiation, both MSCs had similar chondrogenic differentiation capacity, as sulphated glycosaminoglycan content was also similar between MSCs cultured under both conditions (Figure 4(c)).

### 3.6. Gene Expression Profiles

Genome-wide expression profiles of MSCs were analysed by human genome microarray. We analysed the expression patterns of senescent MSCs of the same donor cultured on uncoated and PLL-coated plates. The most significant differentially expressed genes are summarized in Tables 2 and 3. Differentially expressed genes (>twofold) in MSCs cultured on PLL and uncoated plates were sorted into 8 categories according to function: cell adhesion, FGF-2 signalling, cell cycle, oxidative stress, tumorigenicity, stemness, cell differentiation, and cell proliferation.

Upregulated genes included the following: calcium channel, voltage-dependent, L type, alpha 1C subunit (*CACNA1C*), which may play a role in the differentiation of stem cells [31]; delta-like 2 homolog (*DLK2*), a modulator of adipogenesis [32]; nuclear assembly factor 1 homolog (*NAF1*), which is required for telomerase function [33]; centromere protein I (*CENPI*), which is essential for mitosis [34]; apelin (*APLN*) that attenuates oxidative stress [35]; actinin, alpha 2 (*ACTN2*), which is involved in maintaining the cell spreading and motility [36]; ciliary neurotrophic factor receptor (*CNTFR*), which acts on neuronal populations in the developing and mature brain [37]; lethal giant larvae homolog 2 (*LLGL2*), involved in normal cell division [38]; E2F transcription factor 8 (*E2F8*), which plays an important role in the S-phase of the cell cycle [39]; tyrosine kinase, nonreceptor, 2 (*TNK2*), which inhibits the GTPase activity of p21 [40]; inhibin beta B (*INHBB*), which is involved in self-renewal of stem cells [41].

Downregulated genes included the following: hairy/enhancer-of-split related to YRPW motif 1 (*HEY1*), a tumour specific gene [42]; thrombospondin 2 (*THBS2*), which mediates focal adhesion disassembly [43]; leucine rich repeat containing 17 (*LRRC17*), a known negative regulator of osteogenesis [44]; collagen, type XI, alpha 1 (*COL11A1*), which is expressed in tumour cell lines [45]; chitinase 3-like 1 (cartilage glycoprotein-39,* CHI3L1*), which is involved in oxidative stress [46]; sulphates 2 (SULF2), which is upregulated in cancer [47, 48]; neurotrophic tyrosine kinase, receptor, type 2 (*NTRK2*), which stimulates focal adhesion disassembly and is involved in cancer [49, 50].

## 4. Discussion

Adult stem cells (MSCs) have many advantages, such as being less tumorigenic, and they do not trigger immune rejection. MSCs thus hold significant promise for future use in stem cell therapy and tissue engineering. However, there are also disadvantages that currently limit their clinical application. MSCs exist in low quantity from a variety of sources, and it is hard to culture them* in vitro* because they are sensitive to external stimuli and readily enter a state of replicative senescence. The culture and expansion of a large amount of MSCs from primary sources are very important for their successful clinical application. Therefore, the development of an easy and innovative method for cultivating MSCs is critical.

Generally, cells are exposed to complex and highly structured microenvironments regulated by multiple biophysical and biochemical factors such as soluble factors and ECM* in vivo* [51]. The fate of MSCs is controlled by cell to cell and cell to ECM interactions [52]. Similarly,* in vitro* culture conditions have a significant impact on the fate of MSCs with changes in gene and protein expression profiles. ECM proteins constitute a microenvironment that provides structural support and attachment to the cells and offer essential communication between cells and their surrounding environment [53]. In this study, we applied a cell culture condition, in which MSCs were grown on plates coated with PLL, which is known to promote cell adhesion to solid substrates and to recreate the* in vivo* microenvironment. We observed an increase in the number of MSCs cultured on PLL-coated plates with activation of the S-phase of the cell cycle. These results indicate the positive effects of PLL on the proliferation of MSCs in* in vitro* culture. In addition, PLL retarded MSC replicative senescence, as determined by *β*-galactosidase staining, demonstrating that PLL-coated plates provide the necessary microenvironment for optimal growth of MSCs* in vitro*. Interestingly, CD73, ecto-5′-nucleotidase used as a marker for MSCs, was rarely expressed in senescent MSCs, whereas MSCs cultured on PLL-coated plates strongly expressed this marker. It is known that most cells that express CD73 are responsible for the production of extracellular adenosine; however its specific function in MSCs is not fully understood. CD73 may be associated with replicative senescence and be a marker of MSC senescence as indicated by our findings (Figure 3(a)).* Oct4*,* Sox2*, and* Nanog*, which are known pluripotency and stemness markers, are very important for self-renewal of stem cells, and they play an essential role in generating induced pluripotent stem cells [54].* Sox2* is also recently known that it is important in maintenance of cell proliferation and multipotency of MSC [55]. Normally,* Oct4* that is a key transcription factor essential for survival and self-renewal is expressed in adult stem cells. However, the expression of* Oct4* easily disappears during culture* in vitro* [56].* Oct4* was in our study undetectable because genes concerning pluripotency including* Oct4*,* Sox2*, and* Nanog* were analysed in passage 7 MSCs. Our results demonstrated that* Sox2* and* Nanog* are significantly upregulated in MSCs cultured on PLL-coated plates compared with MSCs cultured on uncoated plates. Upregulation of those stemness factors may play a crucial role in increasing MSC proliferation and delaying replicative senescence of MSCs. Moreover, our results suggest that PLL can significantly improve osteogenic differentiation of MSCs. MSCs could not ordinarily differentiate into osteocytes due to cell aggregation and inhibition of proliferation in osteogenic induction environment. However, PLL had no effect on the adipogenic and chondrogenic differentiation capacity of MSCs.

In this study, we present for the first time an analysis of the global gene expression profiles of senescent MSCs cultured on uncoated versus PLL-coated plates, using human genome microarray to gain insight into the molecular characteristics of senescence. Functional classification of differentially expressed genes, according to the Gene Ontology (GO), demonstrated that genes associated with the cell cycle (GO:0007049), cell division (GO:0051301), cell proliferation (GO:0008283), transcription factor activity (GO:0003700), extracellular region (GO:0005576), positive regulation of cell proliferation (GO:0008284), and focal adhesion (GO:0005925) were upregulated in MSCs cultured on PLL-coated plates compared to MSCs cultured on uncoated plates. These findings are in line with the increased proliferation potential of MSCs cultured on PLL-coated plates. Several genes related to cell proliferation might also act as inhibitors of MSC senescence. However, to determine the role of individual genes on MSC, additional studies will be required.

Several studies have reported that MSCs undergo a typical phenomenon of replicative senescence with changing cell morphology and decreasing proliferation under* in vitro* culture. We determined that senescence of bone marrow-derived MSCs occurred after 6 passages. Such senescence is considered to be associated with the accumulation of intracellular reactive oxygen species (ROS) and shortening of telomere length [57]. High levels of ROS are associated with the loss of stemness, and growth arrest is triggered by the p53 and p21 pathways [58, 59]. In this study, we showed that the cell senescence was inhibited by PLL through the suppression of p16^INK4a^ and p21^Cip1^ mRNA expression levels. Based on those mechanisms, it was recently reported that cellular senescence of MSCs could be inhibited via FGF-2 mediated suppression of p53 and p21 [60]. Overall, our study showed that PLL induces the upregulation of genes involved in cell adhesion, FGF-2 signalling, cell cycle regulation, stemness, cell differentiation, and cell proliferation with downregulation of genes associated with oxidative stress and tumorigenicity. Changes in those genes corresponded to significant suppression of MSC senescence by PLL. The genes affected by PLL are summarized in Tables 2 and 3. Our results require further investigation as to the specific genes that have functional implications on senescent MSCs.

Cellular senescence is generally considered an irreversible cellular change. In our study, the proliferation capacity and functionality of senescent MSCs were improved by PLL; however this effect of PLL was reversed when these cells were recultured in the absence of PLL on uncoated plates (data not shown). PLL as a coating substrate affects senescent MSCs when the cells attached on PLL through cell to ECM interaction. Therefore, senescent MSCs on uncoated plates could return to a senescence phenotype if they were not maintained on PLL-coated culture vessels. Previously, Yocum et al. reported that infusing cells labeled with ferumoxides-PLL complex does not affect hematologic or biochemical measures [61]. MSCs cultured on and mixed with PLL may be useful for clinical application because PLL definitely enhances functionality, and it does not alter biochemical and hematologic measurements* in vivo*.

In conclusion, we established an easy and simple culture system for culturing MSCs. Our system enhanced the proliferation rates of MSCs and evokes consistent changes in gene expression associated with MSC stemness markers and differentiation potential. We conclude that senescent MSCs can be converted to a normal-like state using PLL. Improvement of the MSC culture system* in vitro* will contribute greatly to the development of cell-based therapy and regenerative medicine.

## Figures and Tables

**Table 1:** Primer sequences.

Gene	Primer sequence (5′-3′)	Annealing temperature (°C)	Product size (bp)
p16^INK4a^	Forward: CGAATAGTTACGGTCGGAGG Reverse: GCATGGTTACTGCCTCTGGT	62	309
p21^Cip1^	Forward: GCGATGGAACTTCGACTTTG Reverse: CGTTTTCGACCCTGAGAGAGTC	60	285
SH3 (CD73)	Forward: TATTGCACTGGGACATTCGGGT Reverse: GGTTGCCCATGTTGCATTCTCT	62	443
SH2 (CD105)	Forward: CATCCTTGAAGTCCATGTCCTCTT Reverse: GCCAGGTGCCATTTTGCTT	62	95
VCAM-1 (CD106)	Forward: GCTTTCCTGCTCCGAAAATCCT Reverse: AACTGGGCCTTTCGGATGGTAT	62	367
Oct4	Forward: GACAACAATGAGAACCTTCAGGAGA Reverse: TTCTGGCGCCGGTTACAGAACCA	62	218
Sox2	Forward: AACCAAGACGCTCATGAAGAAG Reverse: GCGAGTAGGACATGCTGTAGGT	62	341
Nanog	Forward: ATAGCAATGGTGTGACGCAG Reverse: GATTGTTCCAGGATTGGGTG	62	219
GAPDH	Forward: GTGGTCTCCTCTGACTTCAACA Reverse: CTCTTCCTCTTGTGCTCTTGCT	62	210

**Table 2:** Upregulated genes (>twofold) in MSCs on uncoated and poly-L-lysine- (PLL-) coated plates.

Gene name	Description	NCBI	PLL/UN
Calcium channel, voltage-dependent, L type, alpha 1C subunit	Homo sapiens calcium channel, voltage-dependent, L type, alpha 1C subunit (CACNA1C), transcript	NM_001129827	16.55
Delta-like 2 homolog (*Drosophila*)	Homo sapiens delta-like 2 homolog (*Drosophila*) (DLK2), transcript variant 2	NM_206539	10.97
Nuclear assembly factor 1 homolog (*S. cerevisiae*)	Homo sapiens nuclear assembly factor 1 homolog (*S. cerevisiae*) (NAF1), transcript variant 1	NM_138386	3.82
Centromere protein I	Homo sapiens centromere protein I (CENPI)	NM_006733	3.15
Apelin	Homo sapiens apelin (APLN)	NM_017413	3.07
Actinin, alpha 2	Homo sapiens actinin, alpha 2 (ACTN2)	NM_001103	2.89
Ciliary neurotrophic factor receptor	Homo sapiens ciliary neurotrophic factor receptor (CNTFR), transcript variant 1	NM_147164	2.84
Lethal giant larvae homolog 2 (*Drosophila*)	Homo sapiens lethal giant larvae homolog 2 (*Drosophila*) (LLGL2), transcript variant 3	NM_001031803	2.43
E2F transcription factor 8	Homo sapiens E2F transcription factor 8 (E2F8)	NM_024680	2.23
Tyrosine kinase, nonreceptor, 2	Homo sapiens tyrosine kinase, nonreceptor, 2 (TNK2), transcript variant 2	NM_001010938	2.20
Inhibin, beta B	Homo sapiens inhibin, beta B (INHBB)	NM_002193	2.07

**Table 3:** Downregulated genes (>twofold) in MSCs on uncoated and poly-L-lysine- (PLL-) coated plates.

Gene name	Description	NCBI	PLL/UN
Hairy/enhancer-of-split related with YRPW motif 1	Homo sapiens hairy/enhancer-of-split related to YRPW motif 1 (HEY1), transcript variant 2	NM_001040708	0.43
Thrombospondin 2	Homo sapiens thrombospondin 2 (THBS2)	NM_003247	0.45
Leucine rich repeat containing 17	Homo sapiens leucine rich repeat containing 17 (LRRC17), transcript variant 2	NM_005824	0.47
Collagen, type XI, alpha 1	Homo sapiens collagen, type XI, alpha 1 (COL11A1), transcript variant B	NM_080629	0.47
Chitinase 3-like 1 (cartilage glycoprotein-39)	Homo sapiens chitinase 3-like 1 (cartilage glycoprotein-39) (CHI3L1)	NM_001276	0.48
Sulfatase 2	Homo sapiens sulfatase 2 (SULF2), transcript variant 1	NM_018837	0.48
Neurotrophic tyrosine kinase, receptor, type 2	Homo sapiens neurotrophic tyrosine kinase, receptor, type 2 (NTRK2), transcript variant *c*	NM_001018064	0.50
